# Construction and Properties of Oil-Loaded Soybean Protein Isolate/Polysaccharide-Based Meat Analog Fibers

**DOI:** 10.3390/foods13081159

**Published:** 2024-04-11

**Authors:** Xinyue Zeng, Bing Cui, Di Wu, Jing Li, Hongshan Liang, Bin Zhou, Bin Li

**Affiliations:** 1 College of Food Science and Technology, Huazhong Agricultural University, Wuhan 430070, China; zxywyyx1@163.com (X.Z.); cbtz0817@webmail.hzau.edu.cn (B.C.); qdwdws@126.com (D.W.); lijingfood@mail.hzau.edu.cn (J.L.); lianghongshan@mail.hzau.edu.cn (H.L.); 2 Key Laboratory of Environment Correlative Dietology, Huazhong Agricultural University, Ministry of Education, Wuhan 430070, China; 3 Key Laboratory of Fermentation Engineering, Ministry of Education, National “111” Center for Cellular Regulation and Molecular Pharmaceutics, Hubei Key Laboratory of Industrial Microbiology, School of Biological Engineering and Food, Hubei University of Technology, Wuhan 430068, China; zhoubin4111@163.com; 4 Functional Food Engineering & Technology Research Center of Hubei Province, Wuhan 430070, China

**Keywords:** soybean protein isolate, sodium alginate, rheological property, wet-spinning technique, meat analog fiber

## Abstract

Rationally designing the fibrous structure of artificial meat is a challenge in enriching the organoleptic quality of meat analogs. High-quality meat analog fibers have been obtained by wet-spinning technique in our previous study, whereas introducing oil droplets will further achieve their fine design from the insight of microstructure. Herein, in this current work, oil was introduced to the soybean protein isolate/polysaccharide-based meat analog fibers by regulating the oil droplets’ size and content, which, importantly, controlled the spinning solution characterization as well as structure-related properties of the meat analog fiber. Results showed that the oil dispersed in the matrix as small droplets with regular shapes, which grew in size as the oil content increased. Considering the effect of oil droplets’ size and content on the spinnability of the spinning solution, the mechanical stirring treatment was chosen as the suitable treatment method. Importantly, increasing the oil content has the potential to enhance the juiciness of meat analog fibers through improvements in water-holding capacity and alterations in water mobility. Overall, the successful preparation of oil-loaded plant-based fiber not only mimicked animal muscle fiber more realistically but also provided a general platform for adding fat-soluble nutrients and flavor substances.

## 1. Introduction

As one of the mainstream directions of future food research, plant-based meat analogs have attracted extensive attention [1,2]. Nowadays, the primary research in this area is to simulate structural components (such as meat fibers, adhesives, pigments, mineral elements, etc.), mouthfeel, flavor, and nutrition [3,4]. These factors comprehensively affect consumers’ final acceptance of meat analogs [5,6]. Among them, the simulation of meat fiber structure is the key to achieving the perfect production of meat analog products, which significantly affects the final texture of meat analog products, so it is regarded as a core task in the study of simulated meat [7].

Our previous study has fabricated meat analog fibers utilizing the interaction between protein and polysaccharide and the fast gel properties of polysaccharide via the wet-spinning technique [8]. It effectively avoided the use of harmful chemical solvents and extreme pH conditions, ensured the nutrition and quality of meat fiber, and realized the efficient production of meat analog fibers. However, consumers expect the organoleptic properties of the meat analogs to be the same as that of animal meat [9]. Notably, muscle fibers and intramuscular fat produce these organoleptic properties [10]. In meat, it was reported that the lipids of muscle fibers consist of cytosolic droplets of triacylglycerols and membrane lipids, phospholipids, and cholesterol, whose sizes and contents are related to animal species and growth cycles [10,11]. These cytosolic droplets not only play an important role in the life activities of muscle cells but also contribute to the juiciness, tenderness, and flavor of products [12].

As with meat, it can be assumed that the organoleptic properties of plant-based meat analog fibers can be improved by altering their internal microscopic structure (e.g., adding oil). Nevertheless, it must be considered that the addition of oil not only influences the properties of meat analog fiber but also influences the process conditions. Previous studies [13,14,15] focused on the effect of the fat/fatty acid on high moisture extruded or shear product quality. However, there is still no direct information about the effect of adding oil on the wet-spinning process and the properties of meat analog fiber.

In the current study, the effects of different oil addition methods and oil contents on the spinning medium and fiber properties were investigated. We hypothesize that the addition of oil will increase the water holding capacity but also result in lower spinnability of the fiber. Initially, the soybean oil added to the spinning solution was emulsified by mechanical stirring or high-speed shearing. Subsequently, the distribution of oil droplets in the spinning solution was observed by confocal laser scanning microscopy, and their rheological properties were studied. Furthermore, fibers with different oil contents were fabricated, and their micromorphology, color values, water-holding capacity, ^1^H relaxation time distribution, and mechanical properties were exploited. Overall, this study not only enriched the preparation details of oil-loaded meat analog fiber by wet-spinning technique but also provided a general platform for the addition of fat-soluble nutrients for future application.

## 2. Materials and Methods

### 2.1. Materials

Sodium alginate (SA) powder (Mn = 357,475, Mn/Mw = 1.392, M/G = 0.32) was purchased from Qingdao Hyzlin Co., Ltd. (Qingdao, China). Soybean oil was purchased from a local supermarket (Wuhan, China). Defatted soy flour was supplied by Shandong Yuwang Industrial Co., Ltd. (Yucheng, China). Based on the previous method [16], soybean protein isolate (SPI) was prepared from defatted soy flour. As determined by the Kjeldahl method, the protein content of freeze-dried SPI powder was 90.64 ± 0.37% (N × 6.25). The remaining chemicals were all analytical grades.

### 2.2. Preparation of SPI/SA Oil-Loaded Composite Solution

According to our preliminary experiments, SPI powder was added to stirring distilled water for 2 h to achieve 16 wt% of SPI solutions. Meanwhile, SA powder was dissolved in distilled water to 7 wt%. SA and SPI solutions were kept overnight at 4 °C to hydrate completely. Their pH values were 6.97 and 7.03, respectively.

The preparation method for the SPI/SA oil-loaded composite solution is shown below. Firstly, SPI and SA solutions were blended at equal weights to produce the SPI/SA composite solution (Control). Subsequently, the SPI/SA oil-loaded composite solution was fabricated by blending 1 wt% oil phase (soybean oil) and 99 wt% aqueous phases (SPI/SA composite solution) with different treatments. The oil-loaded composite solution prepared with mechanical stirring treatment at 1000 rpm for 3 min was labeled as MT1, while the oil-loaded composite solution prepared with high-speed shear treatment at 10,000 rpm for 3 min was labeled as HT1. Besides, a series of oil-loaded composite solutions with different oil phases (3, 5, 8, 10, 15 wt%) were prepared by mechanical stirring treatment at 1000 rpm for 3 min, which were labeled as MT3, MT5, MT8, MT10, and MT15, respectively. Finally, a centrifugation at 10,000× *g* for 10 min was conducted to degas the SPI/SA oil-loaded composite solution.

### 2.3. Confocal Laser Scanning Microscopy (CLSM) of SPI/SA Oil-Loaded Composite Solutions

The microstructure of the SPI/SA oil-loaded composite solution was captured by a CLSM (FV3000, Olympus, Tokyo, Japan) using a mixed fluorescent dye solution (1 mg/mL Nile Red and 1 mg/mL fluorescein isothiocyanate (FITC) in alcohol) to mark oil and protein, with an excitation wavelength of 488/575–580 nm (Nile red) and 494/518 nm (FITC) [17]. 100 μL dye solution was blended fully with 5 mL oil-loaded composite solution. Subsequently, the oil-loaded composite solution (30 μL) was dropped on a glass slide and observed at ×40 magnification. The oil phases dyed with the Nile Red are red, whereas the protein phases dyed with the FITC are usually green.

### 2.4. Droplet Size Distribution of SPI/SA Oil-Loaded Composite Solutions

Droplet size distribution of SPI/SA oil-loaded composite solutions was measured with a Mastersizer 2000 (Malvern Instruments Ltd., Malvern, UK). Deionized water was stirred at a speed of 2000 r/min to disperse oil-loaded composite solutions. Dispersant and soybean oil have refractive indexes of 1.33 and 1.47, respectively.

### 2.5. Rheology Measurement of SPI/SA Oil-Loaded Composite Solutions

The steady flow behavior of SPI/SA oil-loaded composite solutions was measured with a DHR-2 rheometer (TA Instruments, Newark, NJ, USA) using a parallel plate geometry (40 mm diameter, 1000 μm gap). At 25 °C, the steady flow behavior of the samples was determined at shear rates ranging from 0.1 s^−1^ to 100 s^−1^. The power-law constitutive equation below [18] can be used to explain flow behavior as follows (Equation (1)):

(1) $$\tau =K\cdot \gamma^{n}$$

where τ is the shearing stress, K is the apparent viscosity constant, γ is the shear rate, and n is the non-Newtonian index.

The spinnability of the composite solutions was estimated using the following formula [19] (Equation (2)):

(2) $$∆\eta =\left(-\mathrm{dlg}\eta |d\gamma^{1/2}\right)\times 10^{2}$$

where Δη is the structural viscosity index.

### 2.6. Physical Stability of SPI/SA Oil-Loaded Composite Solutions

The physical stability of SPI/SA oil-loaded composite solutions was measured by multiple light scattering instruments (TURBISCAN Lab, Formulation, Toulouse, France). Measurements were determined using a pulsed near-infrared LED with a wavelength of 880 nm for 2 h. Meanwhile, the backscattering light from the sample at angles of 180° and 45° was also studied. The physical stability of composite solutions against creaming was evaluated by the change in the Turbiscan Stability Index (TSI).

### 2.7. Preparation of SPI/SA Oil-Loaded Composite Fibers

**Figure 1:**
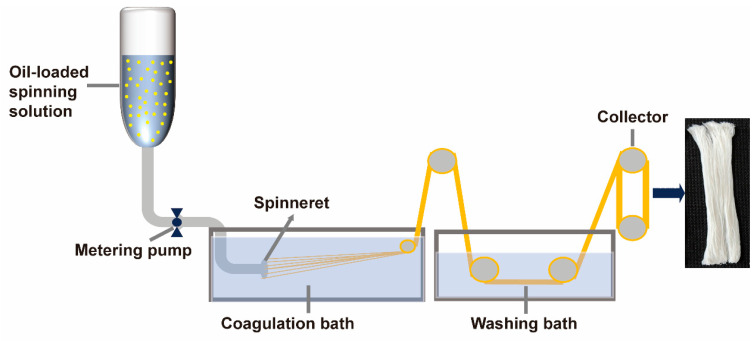
Wet-spinning process flow chart of SA/SPI oil-loaded composite fibers.

The SPI/SA oil-loaded composite fiber was fabricated successfully via the wet-spinning technique. Figure 1 illustrates the process flow for wet-spinning SPI/SA oil-loaded composite fibers. Concretely, a metering pump extruded SPI/SA composite solutions at a speed of 6 mL/min at 25 °C through a 75-hole spinneret (0.12 mm diameter) into a 3% calcium chloride coagulation solution. The SPI/SA oil-loaded composite fibers were then obtained after washing. The control sample fiber was produced with only SPI/SA composite solution.

### 2.8. Microstructures of SPI/SA Oil-Loaded Composite Fibers

#### 2.8.1. Confocal Laser Scanning Microscopy (CLSM)

Protein and Oil droplets were marked with a fluorescent dye solution (1 mg/mL fluorescein isothiocyanate (FITC) and 1 mg/mL Nile Red in alcohol), and then fiber morphology was visualized using a Confocal laser scanning microscopy (FV3000, Olympus, Tokyo, Japan). A volume of 10 μL dye solution was dropped onto the fiber surface and then observed at ×20 magnification. The excitation wavelengths of fluorescent dye were 488 nm (Nile red) and 494 nm (FITC), respectively.

#### 2.8.2. Scanning Electron Microscope (SEM)

Based on the previous method [20], by freezing spun fibers in liquid nitrogen and immediately breaking them, then drying them in a vacuum freeze dryer, accurate cross-sections could be obtained. The surface morphology and cross-sectional structure of freeze-dried fibers after Au sputter coating were studied by SEM (JSM-6390LV, Jeol, Tokyo, Japan) under an accelerating voltage of 10 kV. Recorded all images of 500× magnification.

### 2.9. Color Evaluation of SPI/SA Oil-Loaded Composite Fibers

Colorimeter measurements were performed by a colorimeter (Konica Minolta Business Technologies, Inc., Tokyo, Japan) at six random surfaces of fresh fiber bundles to determine color parameters, such as lightness (L*), redness (a*), and yellowness (b*). A white standard plate (L* = 93.73, a* = 0.07, b* = 2.99) was used to calibrate the colorimeter. The color difference (ΔE) of fiber bundles was calculated by Equation (3) as follows [14]:

(3) $$∆E=\sqrt{{\left(L_{\mathrm{sample}}-L_{\mathrm{standard}*}\right)}^{2}+{\left(a_{\mathrm{sample}}-a_{\mathrm{standard}*}\right)}^{2}+{\left(b_{\mathrm{sample}}-b_{\mathrm{standard}*}\right)}^{2}}$$

### 2.10. Water-Holding Capacity (WHC) of SPI/SA Oil-Loaded Composite Fibers

According to previous studies with some modifications [8], fresh fibers’ water-holding capacity (WHC) was determined to define the fibers’ ability to retain moisture under centrifugal forces. The sample (2 g) was placed into 50 mL tubes and then centrifuged at 2000× *g* for different periods of time (1, 3, 5, 10, 15, and 20 min). Before initial centrifugation, filter paper was used to remove excess moisture on the fresh fibers. Based on the following equation (Equation (3)), the WHC was calculated:

(4) $$\mathrm{WHC}(\%)=\frac{W_{a}}{W_{b}}\times 100$$

In this equation, W_a_ represents fresh fibers’ weight after centrifugation, and W_b_ represents fresh fibers’ weight before centrifugation.

### 2.11. Low-Field Nuclear Magnetic Resonance (LF-NMR) of SPI/SA Oil-Loaded Composite Fibers

^1^H proton signal distribution in fresh fibers was measured with a LF-NMR analyzer (MesoQMR23-060H, Niumag Electric Corporation, Shanghai, China) using the method [21] modified a little bit. Floating water on the fiber bundle surface needed to be removed with filter paper before testing. A 2 g sample was wrapped in polytetrafluoroethylene bags and placed inside a 15-mm diameter glass tube. In this experiment, the analyzer’s operating temperature was 32 °C, and its resonance frequency was 21 MHz. The transverse relaxation time (T2) was determined using the Carr-Purcell-Meiboom-Gill (CPMG) sequence with the following parameters: SW = 100 kHz; RG = 20 dB; NECH = 3000; TE = 0.2 ms; NS = 4, TW = 2000 ms.

### 2.12. Mechanical Analysis of SPI/SA Oil-Loaded Composite Fibers

Since an individual fresh fiber has a similar strength to a muscle fiber, it was not detected by standard texture analyzers and universal extensometers. For this reason, a device that measures the tension of natural muscle fibers was used to test the strength of meat analog fibers [22]. To achieve this, cellulose acetate glue was used to settle a single fresh fiber between force transducers [23]. By using a computer-controlled feedback system (Aurora Scientific 802B, Aurora, ON, Canada), fiber length was rapidly changed. In a sample tank filled with distilled water, four single fibers were measured from each sample. The force response was recorded at each L/Ls (i.e., stretch length/slack length) step after each fiber was stretched intermittently six times. In the statistical analysis, data from fibers were averaged and used as a representative.

### 2.13. The Component Analysis of SPI/SA Oil-Loaded Composite Fibers

The primary composition, including moisture, protein, and total fat, was evaluated using the AOAC standard methods [24].

### 2.14. Statistical Analysis

The results reported (mean ± standard deviation) are based on a minimum of three times of repeated testing. All figures were drawn using Origin 2020b (Originlab Inc., Northampton, MA, USA). IBM SPSS software (version 20.0, IBM Corp., New York, NY, USA) was used to analyze differences between treatments with a *p*-level of 0.05 (Duncan’s test).

## 3. Results and Discussion

According to the literature about muscle fiber [10,11], there are lipid droplets present in animal muscle fibers, which is an important component affecting the tenderness and juiciness of muscles. To mimic meat fibers more realistically, soybean oil was added to the spinning solution to prepare oil-loaded meat analog fibers. Meanwhile, the effects of oil content on the spinning solution behavior were investigated as well.

### 3.1. Effect of Different Emulsifying Methods on Solution Behavior of SPI/SA Oil-Loaded Composite Solution

#### 3.1.1. Microstructures and Droplet size Distribution of SPI/SA oil-Loaded Composite Solution

**Figure 2:**
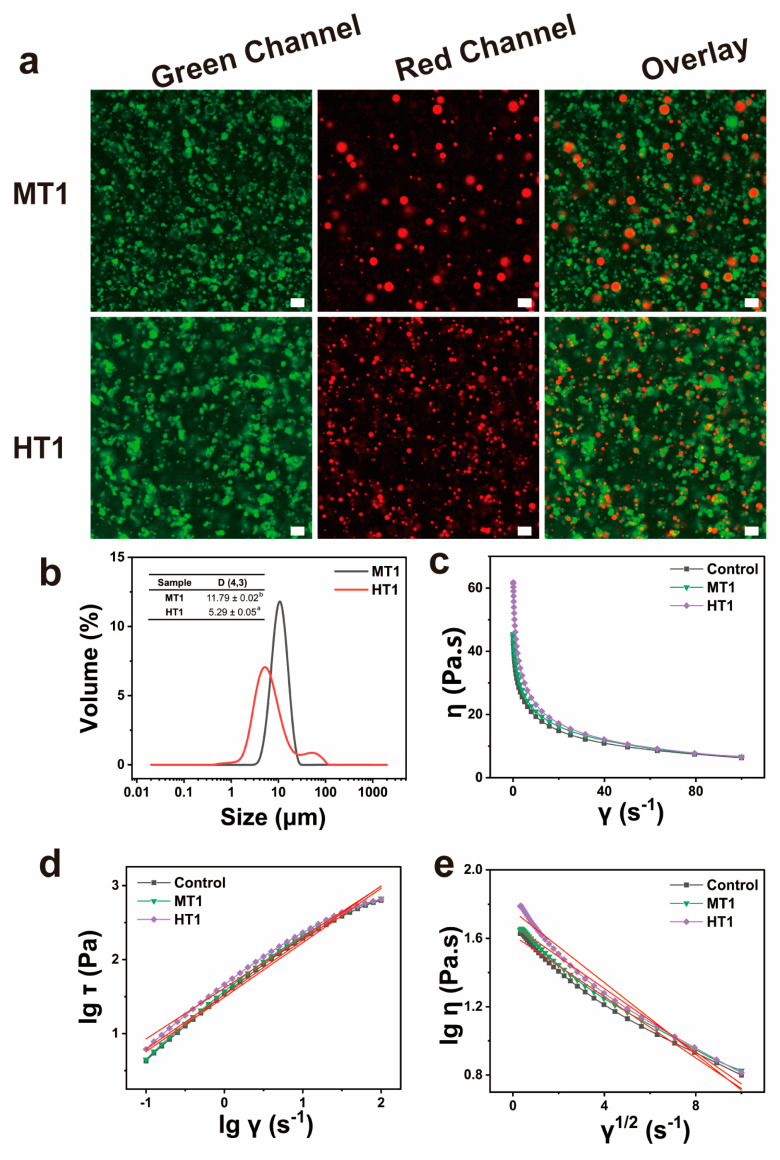
CLSM images (**a**) particle size distribution (**b**) and rheological analysis (**c**–**e**) of SA/SPI composite solutions with 1.0% oil content under mechanical stirring treatment (MT1) and high-speed shear treatment (HT1). Green channel: protein particles (FITC), red channel: oil droplets (Nile Red), scale bar: 20 μm. Different letters (a, b) indicate significant differences at *p* < 0.05.

The CLSM images and particle size distribution of the SPI/SA composite solutions with 1.0% oil content under different treatments are shown in Figure 2a,b. As shown in CLSM, the oil droplets and protein dyed with Nile Red and FITC were presented with green and red, respectively. It was found that the oil droplets uniformly dispersed in the spinning solution. Although the average particle size (D_4,3_) of the emulsion obtained by mechanical stirring treatment (MT1) was twice that of high-speed shear treatment (HT1), the particle size distribution of that (MT1) was narrow, indicating that the oil droplets of that (MT1) were more uniform in size.

#### 3.1.2. Rheological Properties of SPI/SA Oil-Loaded Composite Solution

Figure 2c–e revealed the effect of different treatments on the rheological properties of the SPI/SA oil-loaded composite solutions, which were a typical shear thinning and pseudoplastic fluid (Figure 2c). The non-Newton index (n) of them could be fit by Figure 2d and calculated according to Equations (1) and (2). From Table 1, a shear-thinning nature (<1) was shown again. Besides, the non-Newton index values of the Control and MT1 were similar, indicating that the oil droplets added by mechanical stirring treatment had no significant effect on the spinning solution. On the contrary, the non-Newton index value of HT1 was lower than the Control, implying the oil droplets added by high-speed shear treatment decreased the fluidity of the spinning solution. This might be due to the interaction between proteins and polysaccharides on the interface of the oil droplets. Compared with MT1, HT1 has a smaller oil droplet size, resulting in a larger surface area, and thus a stronger interaction occurred with polysaccharides. The structural viscosity index (Δη) was calculated from Figure 2e and Equation (2), which characterized the spinnability of the spinning solution. It is generally true that the higher the structural viscosity index, the worse the spinnability [19]. As can be seen in Table 1, HT1 had a significantly higher structural viscosity index than Control and MT1, indicating that MT1 was better suited for spinning than HT1. Hence, the mechanical stirring treatment was chosen for the following tests.

### 3.2. Characterization of SPI/SA Oil-Loaded Composite Solution

#### 3.2.1. CLSM Analysis

**Figure 3:**
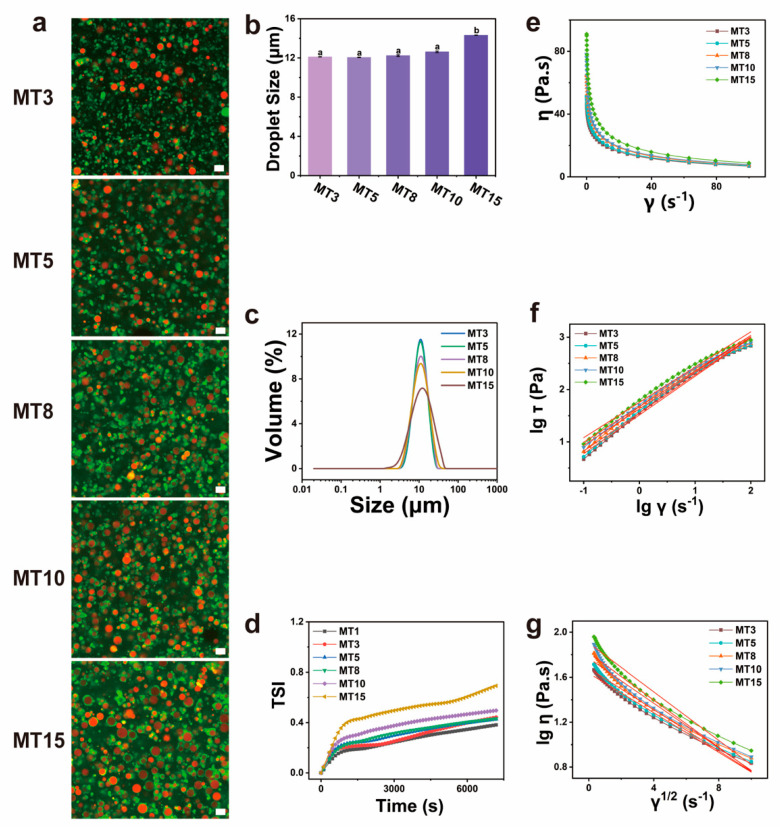
CLSM images (**a**), droplet size (**b**) and volume (**c**), TSI (Turbiscan stability index) curves (**d**), rheological analysis (**e**–**g**) of different SA/SPI composite solutions MT1, MT3, MT5, MT8, MT10, MT15 with different oil contents (1, 3, 5, 8, 10, 15 wt%) under mechanical stirring treatment. Scale bar: 20 μm. Different letters (a, b) indicate significant differences at *p* < 0.05.

The CLSM images of the SPI/SA composite solutions with different oil contents under mechanical stirring treatment are shown in Figure 3a. The soybean oil was stained with Nile Red (Red), while protein particles were marked by FITC (Green). The CLSM images confirmed that soybean oil droplets were coated around the circle of protein particles, and the number and distribution density of oil droplets increased with the oil content increased.

#### 3.2.2. Droplet Size and Distribution

Figure 3b,c displayed the mean droplet diameters (D_4,3_) increased with an increase in oil content, and droplet size distribution became wide, which attributed that the emulsifying ability of a certain amount of protein was inversely proportional to the oil content, and excess oil led to the aggregation of oil droplets.

#### 3.2.3. Stability Evaluation

The values of the turbiscan stability index (TSI) usually represent the emulsion’s overall stability, which was a non-destructive method [25]. There was a greater variation in droplet concentration with a higher TSI, and the emulsion was less stable [26]. As displayed in Figure 3d, the TSI increased with the increase of oil contents, which was ascribed to the emulsifiability of the composite spinning solution, indicating that the higher the oil content, the more unstable the emulsion. The TSI was less than 1.0 in this study (TSI < 0.5 indicates the emulsion is very stable; TSI < 1.0 indicates the emulsion was unstable at the beginning). Therefore, the emulsions with an oil content of less than 10% were stable, which ensured the stability of the spinning solution during the spinning process.

#### 3.2.4. Rheological Properties

Figure 3e–g revealed the rheological properties of the SPI/SA composite solution with different oil contents. From Figure 3e, the SPI/SA composite solution’s viscosity decreased with increasing shear rate, indicating that the composite solution was typical shear thinning and pseudoplastic fluid. According to Figure 3f,g, and Table 2, Δη increased while n values decreased with an increase in oil content, which indicated that high oil contents were unfavorable for spinning. The results were attributed to the increase in steric hindrance caused by the increased number and size of oil droplets [27].

### 3.3. Properties of SPI/SA Oil-Loaded Composite Fibers

As widely acknowledged, meat analog fiber’s performance plays an important role in determining meat analog food quality. Based on this, the SPI/SA oil-loaded composite fibers were obtained using the wet-spinning technique, and their microstructures, color, and other physicochemical properties were evaluated in detail.

#### 3.3.1. Microstructures Analysis

**Figure 4:**
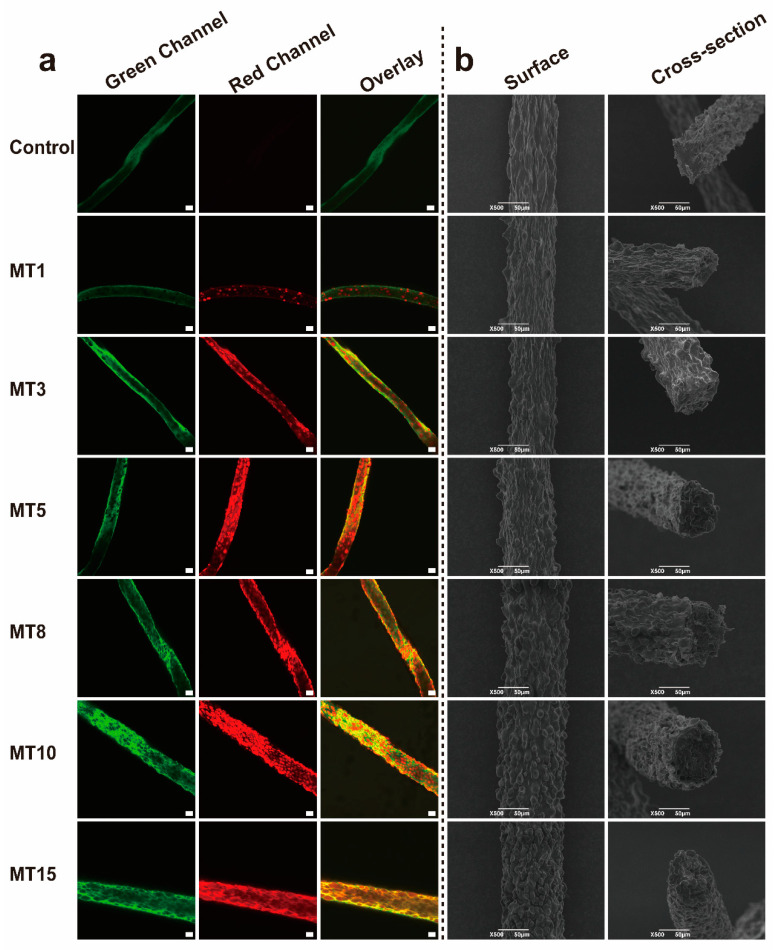
CLSM (**a**) and SEM (**b**) images of different SA/SPI oil-loaded composite fibers (Control: 0%, MT1: 1%, MT3: 3%, MT5: 5%, MT8: 8%, MT10: 10%, MT15: 15% wt%). Green channel: protein particles (FITC), red channel: oil droplets (Nile Red), scale bar: 50 μm.

CLSM images (Figure 4a) displayed the distribution of oil droplets in the fibers, proving that the oil-loaded plant-based fibers were fabricated successfully. The number and size of oil droplets in the fiber also increased as the oil contents rose, which was consistent with the CLSM results of the spinning solution. The surface and cross-sectional morphology of the fibers were characterized by SEM (Figure 4b). As the oil content increased, the more convex the surface was, the more oil droplets were coated by the fibers. In addition, fiber diameter increased with increasing oil content, possibly due to an increase in spinning solution viscosity.

#### 3.3.2. Color Changes

**Figure 5:**
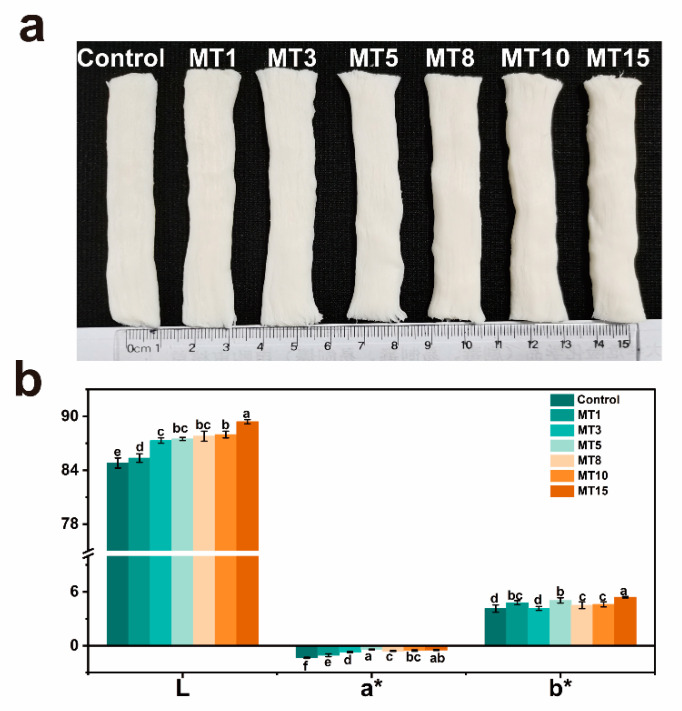
The appearance (**a**) and color values (**b**) of different SA/SPI oil-loaded composite fibers (Control: 0%, MT1: 1%, MT3: 3%, MT5: 5%, MT8: 8%, MT10: 10%, MT15: 15% wt%) Different letters (a, b, c, d, e, f) indicate significant differences at *p* < 0.05. Error bars show standard deviation.

The acceptance of consumers would be affected by color as an organoleptic characteristic. Color attributes (Figure 5) were adopted to quantify fresh fiber bundle appearance and indirectly reflect chemical reaction levels. Adding soybean oil significantly increased composite fibers’ lightness, which was directly related to light reflection caused by oil droplets. The addition of high oil content (MT15) resulted in the highest L* value (89.40 ± 0.23) and the lowest a* value (−0.48 ± 0.05). This observation was in line with those in the previous report [14] that the addition of linoleic acid in pea protein isolate (PPI) extrudates caused a higher L* value and lower a* value than the control. Furthermore, the result showed that oil caused a significant decrease in the ΔE values from 9.12 to 4.99~8.64, probably because of the oil-containing small droplets giving rise to a smoother appearance (see Figure 5a).

#### 3.3.3. Water Holding Capacity

**Figure 6:**
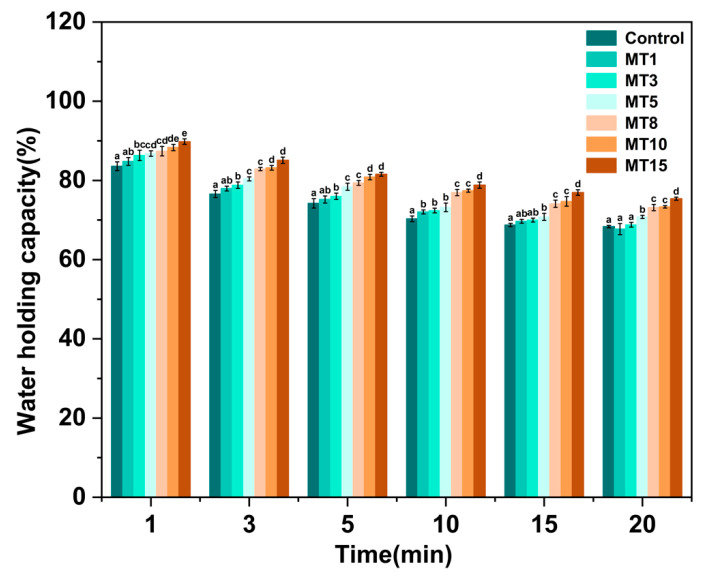
The water-holding capacity of different SA/SPI oil-loaded composite fibers (Control: 0%, MT1: 1%, MT3: 3%, MT5: 5%, MT8: 8%, MT10: 10%, MT15: 15% wt%). Different letters (a, b, c, d, e) indicate significant differences at *p* < 0.05. Error bars show standard deviation.

The juiciness of meat and meat products is influenced by their moisture content and water-holding capacity [28,29], which is a key factor affecting meat products’ quality. The results in Figure 6 indicated that the increment of oil content resulted in higher WHC of the composite fibers, which was attributed to the fact that the oil droplets strengthened capillary forces to hold water in the gel network of the fibers [30]. On the other hand, Table 3 showed that liquid oil replaced part of the water, and the total amount of oil and water was higher in oil-loaded fibers than that in Control, resulting in a decrease in centrifugal water loss rate and an increase in WHC. As a result, adding oil to meat analog fibers is beneficial for keeping their juiciness.

#### 3.3.4. Low-Field NMR Spin-Spin Relaxation (T_2_) Measurements

**Figure 7:**
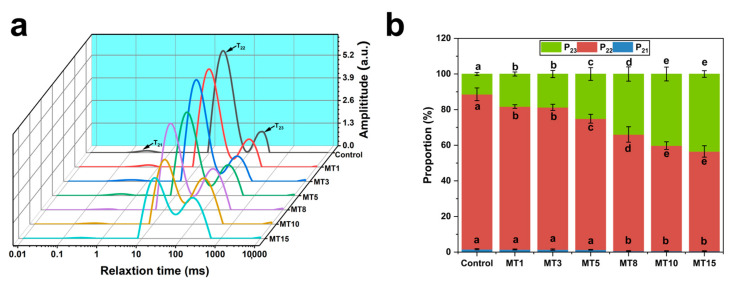
Distributed T_2_ relaxation times (**a**) and proportions of ^1^H proton signal distribution (**b**) of SA/SPI oil-loaded composite fibers (Control) and series of oil-loaded composite solutions MT1, MT3, MT5, MT8, MT10, MT15 with different oil phases (1, 3, 5, 8, 10, 15 wt%). Different letters (a, b, c, d, e) indicate significant differences at *p* < 0.05. Error bars show standard deviation.

The mobility of water and oil in fibers was investigated by ^1^H NMR relaxometry, as displayed in Figure 7a,b. In fibers, T_21_, T_22_, and T_23_ are the transverse relaxation times with different states of proton signals (^1^H), which corresponds to the mobility of water or oil components [31]. Their corresponding area fractions are P_21_, P_22_, and P_23_ [32,33]. With the addition of oil to the fibers, there was some superposition of proton signals between T_22_ and T_23_. Meanwhile, P22 decreased, whereas P_23_ increased. This states that the addition of oil increased the mobility of water or oil components [34]. Combined with Table 3, it was found that the moisture decreased with increasing oil content in fibers, which indicated the mobility of proton signals in oil was superior to that in water [31]. The decrease in P_22_ might be caused by a decrease in the amount of water binding to the protein [34]. P_23_ increased after oil was added, indicating superior mobility of water or oil, which might be an advantage for improving fiber juiciness.

#### 3.3.5. Mechanical Property

**Figure 8:**
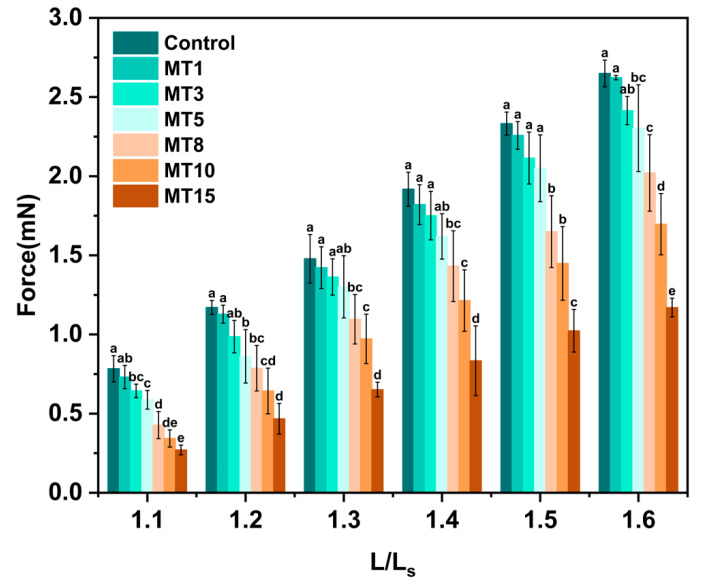
The tensile strength of different SA/SPI oil-loaded composite fibers (Control: 0%, MT1: 1%, MT3: 3%, MT5: 5%, MT8: 8%, MT10: 10%, MT15: 15% wt%). Different letters (a, b, c, d, e) indicate significant differences at *p* < 0.05. Error bars show standard deviation.

Mechanical property is an essential aspect of any material and directly influences its application. As shown in Figure 8, the fiber’s tensile strength decreased as the oil content increased, especially when the oil content exceeded 3%. It might be because of an increase in oil content, which led to larger size and aggregations of oil droplets in the spinning solution and fibers (see Figure 3 and Figure 4), hindering the interaction of SPI/SA and the cross-linking of SA and Ca^2+^.

## 4. Conclusions

In this study, the effect of oil droplet size and oil contents on the solution behavior of SPI/SA composite systems and the properties of meat analog fibers processed by the wet-spinning technique were investigated. As a result of analyzing the relationship between oil droplet size and the solution spinnability, mechanical stirring was selected as the treatment method. The findings from confocal laser scanning microscopy (CLSM) and scanning electron microscopy (SEM) revealed a consistent pattern in the dispersion of oil droplets within the matrix, both pre-and post-spinning, with a discernible increase in droplet size corresponding to increased oil concentrations. Concurrently, the increase in oil content correlated with decreased spinnability of the spinning solution, with a threshold observed at 10% oil content, beyond which destabilization of the spinning solution was evident. The incorporation of oil into fibers increased its brightness. Remarkably, the increase in oil content amplified the water-retention capacity while modifying water mobility within meat analog fibers, albeit at the expense of mechanical integrity. Consequently, producers are urged to meticulously calibrate oil content to optimize the balance between its impact on the spinning process and the resultant enhancement of fiber properties in the practical manufacturing of plant-based meat analogs.

## Figures and Tables

**Table 1:** The Non-newtonian (n) and Structural viscosity index (Δη) of SA/SPI composite solution without oil (Control) and with 1.0% oil content under mechanical stirring treatment (MT1) and high-speed shear treatment (HT1).

Sample	n	R_1_^2^	Δη	R_2_^2^
Control	0.7353	0.99	8.97	0.98
MT1	0.7352	0.99	9.06	0.98
HT1	0.6878	0.99	10.49	0.97

**Table 2:** The Non-newtonian (n) and Structural viscosity index (Δη) of SA/SPI composite solution with different oil contents (MT3, MT5, MT8, MT10 and MT15) under mechanical stirring treatment.

Sample	n	R_1_^2^	Δη	R_2_^2^
MT3	0.7324	0.99	9.117	0.98
MT5	0.7272	0.99	9.190	0.98
MT8	0.7010	0.99	9.976	0.97
MT10	0.6802	0.99	10.51	0.95
MT15	0.6741	0.99	10.82	0.96

**Table 3:** The component of SA/SPI composite fibers without (Control) and with different oil contents (MT1, MT3, MT5, MT8, MT10, and MT15).

Sample	Moisture (%)	Protein (%)	Oil (%)
Control	74.30 ± 0.89 ^a^	15.50 ± 0.46 ^a^	0.11 ± 0.05 ^a^
MT1	72.84 ± 0.54 ^a^	14.59 ± 0.32 ^a^	1.51 ± 0.08 ^a^
MT3	70.67 ± 1.20 ^b^	13.29 ± 0.65 ^b^	5.06 ± 0.12 ^b^
MT5	68.46 ± 0.68 ^c^	13.18 ± 0.48 ^b^	7.81 ± 0.35 ^c^
MT8	63.31 ± 0.76 ^d^	13.03 ± 0.63 ^b^	13.72 ± 1.02 ^d^
MT10	57.98 ± 0.57 ^e^	12.74 ± 0.53 ^b^	17.84 ± 0.89 ^e^
MT15	55.20 ± 0.43 ^f^	10.87 ± 0.62 ^c^	24.71 ± 1.32 ^f^

Different letters (a, b, c, d, e, f) indicate significant differences at *p* < 0.05.

## Data Availability

The original contributions presented in the study are included in the article, further inquiries can be directed to the corresponding author.
